# Exploring Public Perspectives on Organ Donation in Makkah, Saudi Arabia

**DOI:** 10.7759/cureus.106992

**Published:** 2026-04-13

**Authors:** Khalid Basamih, Omar Ba Mhel, Mohammed Alhazmi, Muhannad Alharbi, Zayed Alharbi, Saleh Al-Elyani, Fouz Alotibe, Nada Bajuaifer

**Affiliations:** 1 Department of Medicine and Surgery, College of Medicine, Umm Al-Qura University, Makkah, SAU; 2 Department of Hematology and Immunology, College of Medicine, Umm Al-Qura University, Makkah, SAU

**Keywords:** beliefs, makkah, organ donation, perspectives, religion

## Abstract

Background

Organ donation involves giving organs to help others in need of transplants. It can be carried out while the individual is still alive or after death (e.g., kidney donation). Strict legal requirements and moral principles govern this life-saving action. Because supply is limited and demand is strong, awareness and registration are essential. In Saudi Arabia, despite religious rulings (fatwa) permitting organ donation since 1982, significant gaps persist between transplant demand and donor availability.

Objectives

This cross-sectional study aimed to evaluate knowledge, attitudes, and barriers regarding organ donation among adults in Makkah.

Methods

A cross-sectional study was conducted online using Google Forms (Google, Inc., Mountain View, CA) from 04 December to 30 December 2024 to investigate public perspectives on organ donation in Makkah, Saudi Arabia. A convenience sampling strategy was employed, and the questionnaire was distributed via various social media channels, including among adult residents of Makkah aged 18 years or older. Age eligibility was verified by self-report, with responses from individuals under 18 excluded. Associations between categorical variables were assessed using chi-squared tests, with effect sizes reported using Cramer's V.

Results

The study surveyed 1,184 residents of Makkah to assess public perspectives on organ donation. Most respondents were young (58.1%, n = 688 aged 18-25), female (65.2%, n = 772), and Saudi (94.7%, n = 1,121), with nearly half holding university degrees (47.1%, n = 558). Despite the high willingness to donate to family or friends (72.7%, n = 861), only 8.2% (n = 97) had an organ donor card, and 6.4% (n = 76) had formally signed one. The internet was the primary source of information (45.4%, n = 538), while knowledge about donation eligibility was variable: most correctly identified kidney (63.7%, n = 754) and liver (53.4%, n = 632) as living-donor organs, but some incorrectly believed the heart (4.9%, n = 58) or brain (3.9%, n = 46) could be donated by living donors. Religious beliefs influenced 43.3% (n = 513) of participants, and 29.4% (n = 348) reported beliefs that hinder donation. Most prioritized donating to the sickest patients (87.7%, n = 1,038) and younger recipients. Willingness to donate (defined as possessing or signing a donor card) was significantly associated with knowing a living organ donor (chi-squared test, p < 0.001, Cramer's V = 0.153), absence of religious barriers (p = 0.002, V = 0.099), and perceived personal importance of donation (p < 0.001, V = 0.136). Nearly all participants (91.7%, n = 1,086) agreed that the public needs more information about organ donation.

Conclusion

This study reveals a significant gap between expressed willingness to donate (72.7%) and actual donor registration (8.2%). The strongest predictors of positive donation attitudes were personal connections to organ donation (14.3% vs. 5.8% willing among those with vs. without knowing a living donor, p < 0.001) and perceived personal importance. Public health interventions should focus on converting positive attitudes into action through simplified registration processes and continued engagement with religious scholars to address misconceptions.

## Introduction

Organ donation and transplantation are life-saving therapy that addresses end-stage organ failure, yet it faces a persistent global imbalance between organ supply and demand. This option can enhance the quality of life and facilitate a return to personal and professional activities [1]. Organ donation refers to the donation of biological tissue or an organ from a living or deceased person to a recipient in need of a transplant. Organ transplantation is the preferred mode of replacement therapy and can be the last resort for lifesaving or life-improving treatments [2].

The global demand for organ transplantation far exceeds the supply, resulting in a considerable imbalance between the number of patients on waiting lists and the available organs for transplantation [3,4]. It has been documented that the organs from a single donor can save or help up to as many as 50 people. However, this figure represents an upper theoretical estimate, and the actual number of recipients is often lower due to clinical and logistical constraints [2]. There are three types of organ donors: the living donor, donors after circulatory death (DCDs), and donors after brain death. There are conditions where organ donation is ruled out completely, such as the donor diagnosed with cancer, an untreated infection, or a condition that affects the nervous system [5]. However, eligibility for organ donation may vary depending on clinical evaluation and established medical guidelines. [3,4]

However, attitudes and perceptions regarding organ donation are still complicated in Saudi Arabia and are frequently impeded by logistical, cultural, and religious barriers. Saudi Arabia represents a permissive environment for organ donation, supported by early Islamic fatwas and the establishment of national organ transplantation programs. The first organ transplant in Saudi Arabia was renal transplantation performed in 1979 [6]. The term “fatwa” in Islam refers to a legal ruling or interpretation issued by a recognized religious authority. In the context of organ donation, the first Islamic Council of Saudi Arabia issued a resolution in 1982 permitting the use of organs from both living and deceased donors for transplantation [7]. Many studies have assessed public knowledge and attitudes toward organ donation. From an Islamic perspective, a 1996 survey of 223 males found that while 67% were willing to donate, only 56% were aware of the Islamic legislation (fatwa) on organ donation [8]. Another study, conducted in 2005, showed that an increase in people’s understanding of the fatwa is correlated with higher levels of education [9]. A more recent study, conducted in 2017, on adults in Al-Kharj, Saudi Arabia, found that 97% of participants were unaware of where to register as organ donors, and 35.6% were unaware that organ donation was legal in Saudi Arabia. Similar levels of limited awareness and persistent hesitancy toward organ donation continue to be reported in Saudi Arabia, as shown in previous local studies [10]. These findings highlight significant gaps in public awareness, despite the legal and religious frameworks that support organ donation. However, notable gaps exist in the existing literature. Makkah is a unique sociocultural and religious core in Saudi Arabia, distinguished by its great religious significance, a diversified resident population, and constant exposure to religious messaging as a result of pilgrims’ presence throughout the year. Despite these distinguishing qualities, few studies have specifically studied public attitudes on organ donation in this setting, with most previous studies focusing on other parts of the country. Understanding views against organ donation in Makkah is thus critical for establishing culturally sensitive and successful awareness campaigns. As a result, the purpose of this study is to investigate public perceptions of organ donation in Makkah, with an emphasis on knowledge, attitudes, and awareness.

## Materials and methods

Study design

A community-based cross-sectional survey was conducted to investigate public perspectives on organ donation in Makkah, Saudi Arabia. The study questionnaire addressed socio-demographic characteristics, donation preparedness and legal documentation, donation knowledge and beliefs regarding eligibility and procedures, social exposure and information sources, religious and personal beliefs, transplant prioritization and ethical considerations, as well as attitudes toward organ donation and the medical system. A copy of the questionnaire was provided in the Appendices. The questionnaire was distributed online through various social media platforms. A non-probability convenience sampling method was used, and participation was voluntary among adult residents of Makkah. The study was conducted after approval from the Umm Al-Qura University Institutional Research Board.

Study population and sample size calculation

The study population included adult residents of Makkah aged 18 years or older. The inclusion criteria were adults aged ≥18 years residing in Makkah, irrespective of gender. The exclusion criteria included individuals younger than 18 years, non-residents of Makkah, and participants with incomplete responses, those who declined to participate, or were visitors to Makkah. Sample size calculation was performed using the Raosoft sample size calculator (Raosoft, Inc., Seattle, WA), which was based on an estimated population of approximately 2,427,924 in Mekkah [11,12].

A sample size of 385 participants was needed. However, a larger sample (n = 1,184) was obtained to increase statistical power and improve the precision of the estimates.

Questionnaire development and structure

The questionnaire was organized into several sections. The first section captured socio-demographic data, including items on residence in Makkah, age, gender, nationality, and education level. The following section focused on donation preparedness and legal documentation. Next, the questionnaire addressed donation knowledge and beliefs regarding eligibility and procedures. Another section examined social exposure and information sources, asking whether participants knew anyone who had received a tissue or organ transplant, had been a living organ donor, or knew someone in need of a transplant, along with the primary source of information on organ donation. The questionnaire also included a section on religious and personal beliefs. The final section addressed transplant prioritization and ethical considerations by asking participants to indicate their views on the fairness of transplant allocation, including factors such as age, smoking status, waiting list position, and sickness level. The section also assessed the overall attitudes toward organ donation and trust in the medical system.

The survey was modeled after Lee et al. [13]. The questionnaire was originally developed in English and was translated into Arabic with clear wording to ensure suitability for the study population. The translated version was appropriate for the local community and sufficient to fulfill the objectives of the study.

Data collection and management

Responses were collected using the Google Forms platform (Google, Inc., Mountain View, CA) [14]. Responses were automatically recorded and securely stored in a digital format. A unique coding system was used to anonymize the data, ensuring that no personal identifying information was included in the dataset. Only the research team had access to the raw data, and all responses were managed in accordance with established data protection protocols. Due to the use of online convenience sampling, there is a possibility of selection bias, particularly the underrepresentation of individuals without internet access or those not active on social media.

Ethical considerations

The study was conducted following approval from the Umm Al-Qura University Institutional Research Board. A statement was included on the cover page of the survey, explaining the objectives of the study, the voluntary nature of participation, and the confidentiality of responses. Participants were informed that no personal identifying information would be collected and that all data would be used solely for research purposes. The statement emphasized that participation was entirely voluntary, and respondents had the right to withdraw at any time without providing a reason. By proceeding with the questionnaire, participants confirmed their consent to participate in the study. Data access was restricted to the research team, and all collected data were stored securely in compliance with ethical guidelines.

Statistical analysis

Descriptive statistics were performed using counts and percentages for categorical variables. Continuous variables were presented as means ± standard deviations or as medians with ranges, depending on the distribution. Group comparisons were performed using Student’s t-test or the Mann-Whitney U test for continuous normal and non-normal/ordinal variables, respectively. The chi-square test was used to assess the associations between categorical variables. The willingness to donate was identified as either having a donor card or having signed a donor card, with the distinction made to explore the factors influencing willingness to donate. Owning a donor card reflects initial intent; while signing it represents formal, legal consent. This distinction was important before analysis was performed to identify factors associated with the willingness to donate. Independent variables included demographic characteristics, perceptions, and attitudes toward organ donation. Cramer’s V was used to evaluate the strength of the association, as it is useful when comparing variables with different numbers of categories. Effect sizes for Cramer’s V can be interpreted as small (0.1), medium (0.3), or large (0.5). Hypothesis testing was performed at 5% level of significance [15].

All statistical analyses were performed using R software (version 4.3, R Foundation for Statistical Computing, Vienna, Austria), which is free and open-source software available under the GNU General Public License. The following R packages were used: base R for descriptive statistics and chi-square tests; all packages used are freely available from the Comprehensive R Archive Network (CRAN).

## Results

Descriptive statistics and social exposure to organ donation

The questionnaire was completed by 1,184 respondents (Table 1). Among these, two-thirds were female (n = 772, 65.2%). Respondents aged 18-25 years represented more than one-half (n = 688, 58.1%), followed by 36-45 years (n = 161, 13.6%) and 26-35 years (n = 127, 10.7%). All participants resided in Mekkah, with most being Saudi nationals (n = 1,121, 94.7%). More than one-half of respondents completed university education (n = 558, 47.1%), and more than one-third completed only high school (n = 455, 38.4%). Only 97 respondents (8.19%) had an organ donor card, and only 76 (6.42%) had signed one. A small number reported having a living will (n = 117, 9.88%), with the majority (n = 1,067, 90.1%) not having one. Family discussions regarding organ and tissue donation were uncommon, as 979 (82.7%) respondents indicated that their family members were unaware of their wishes.

**Table 1:** Demographic characteristics, donation preparedness, and social exposure to organ donation ^¶^ Yes/no questions. The n (%) who responded with “yes” is shown.

	Overall
	N = 1,184
Gender	
Female	772 (65.2%)
Male	412 (34.8%)
Age	
18-25	688 (58.1%)
26-35	127 (10.7%)
36-45	161 (13.6%)
46-55	147 (12.4%)
56-65	53 (4.48%)
> 65	8 (0.68%)
Nationality	
Non-Saudi	63 (5.32%)
Saudi	1,121 (94.7%)
Education	
Middle school	15 (1.27%)
High school	455 (38.4%)
Diploma	91 (7.69%)
Primary school	5 (0.42%)
University	558 (47.1%)
Post-graduate	60 (5.07%)
Have an organ donor card ^¶^	97 (8.19%)
Signed organ donor card ^¶^	76 (6.42%)
Have a living will ^¶^	117 (9.88%)
Family members aware of wishes regarding organ and tissue donation in case of inability to make decisions ^¶^	205 (17.3%)
Social exposure	
Know anyone who has received a tissue transplant ^¶^	127 (10.7%)
Know anyone who has received an organ transplant ^¶^	433 (36.6%)
Know anyone who was a living organ donor ^¶^	391 (33.0%)
Know anyone who needs an organ or tissue transplant ^¶^	275 (23.2%)
Consider being a living organ donor if a family member or close friend needed a transplant ^¶^	861 (72.7%)
Ever discussed the topic of organ donation with family members ^¶^	461 (39.0%)

**Figure 1:**
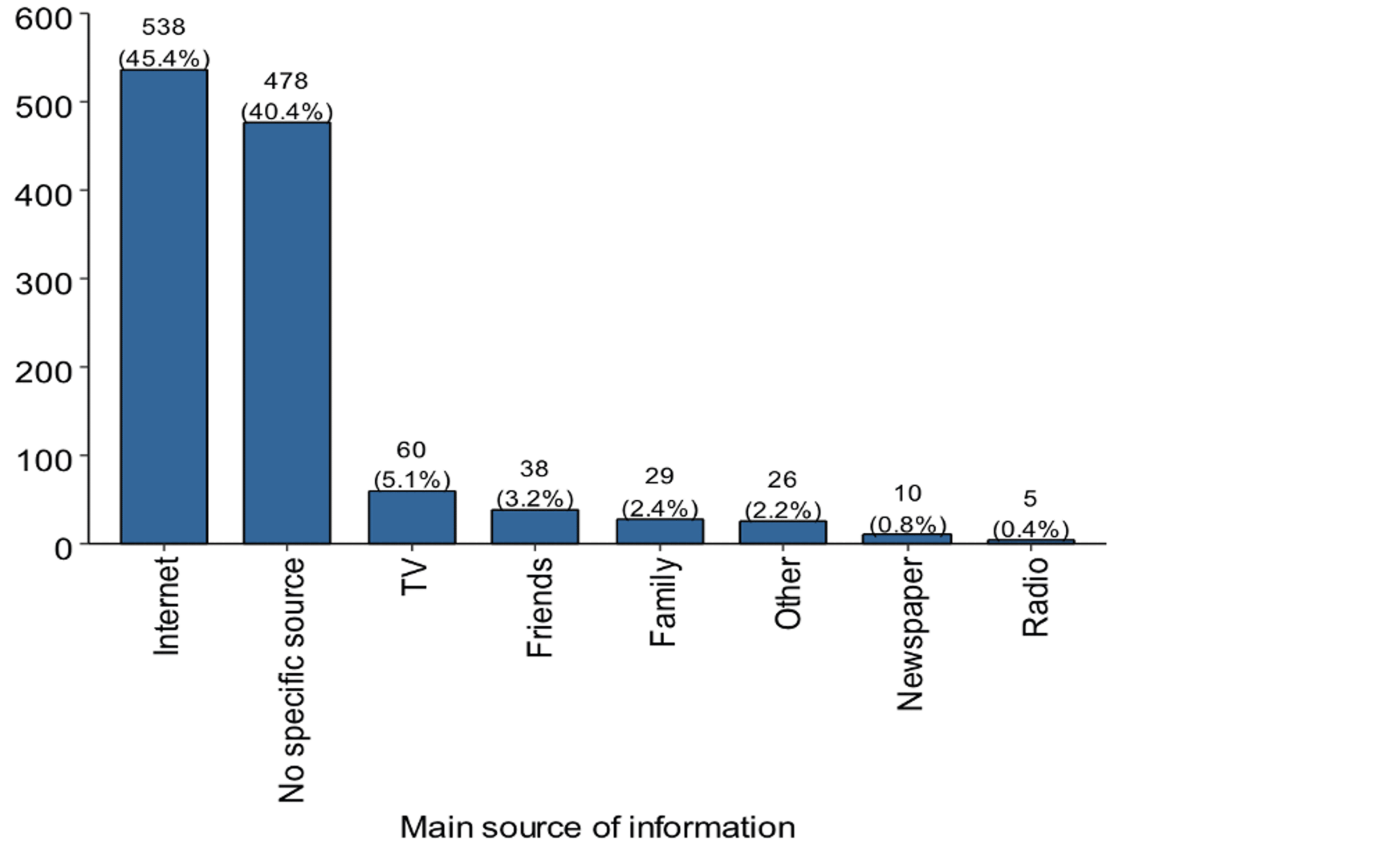
The main source of information regarding organ donation Image generated using R software (version 4.3, R Foundation for Statistical Computing, Vienna, Austria).

The internet was the most commonly reported source (n = 538, 45.4%), followed by those who reported no specific source (n = 478,40.4%). Traditional media sources, such as TV, were reported by 60 (5.1%) participants, while friends and family were reported by 38 (3.2%) and 29 (2.4%) participants, respectively (Figure 1). Other sources made up 26 (2.2%).

Donation knowledge and beliefs about donation eligibility and procedures

**Figure 2:**
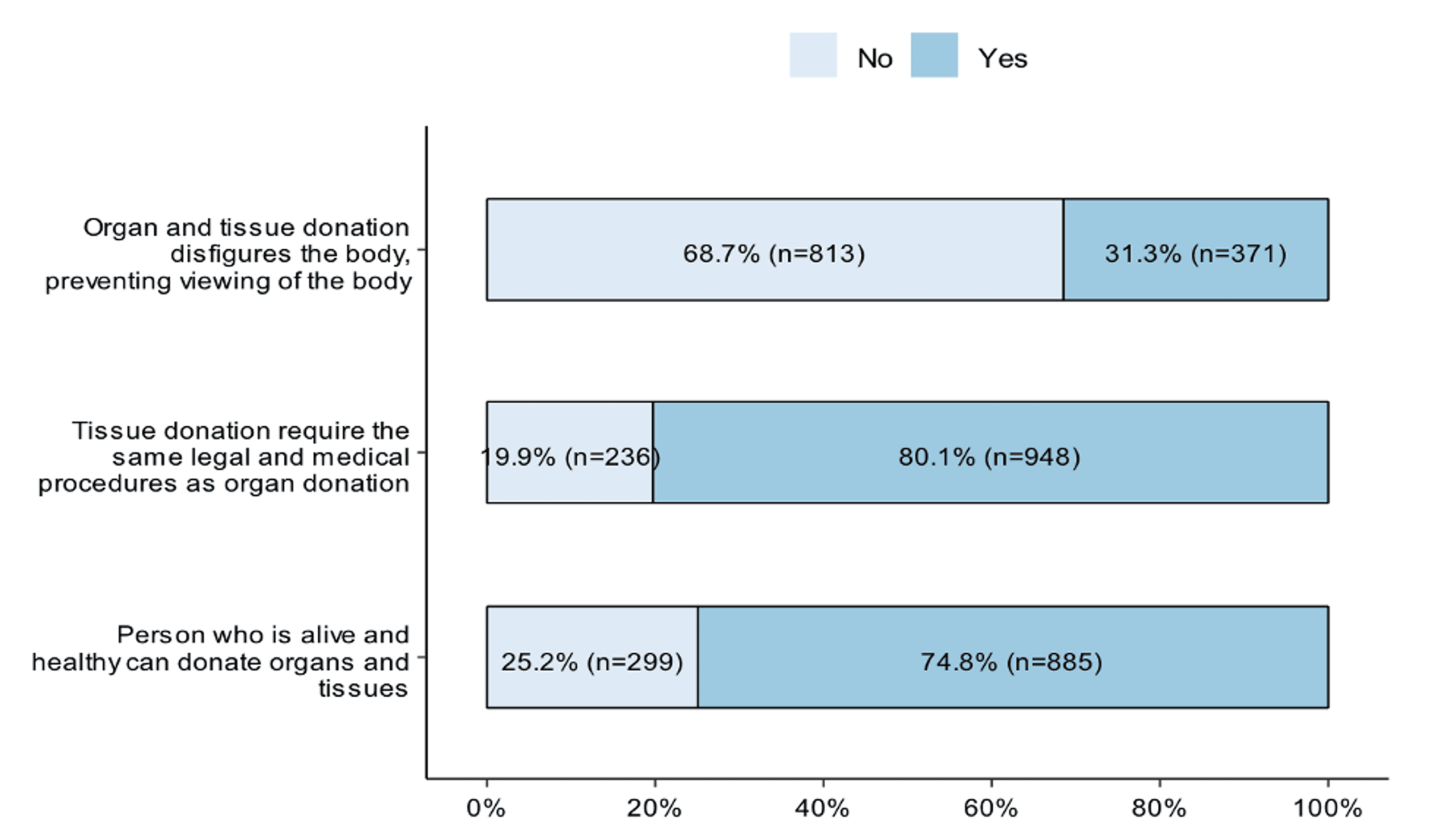
Knowledge and beliefs about donation eligibility and procedures Image generated using R software (version 4.3, R Foundation for Statistical Computing, Vienna, Austria).

The results indicated varying levels of knowledge regarding organ and tissue donation. As shown in the figure, 813 (68.7%) participants correctly disagreed with the misconception that organ and tissue donation disfigured the body. Regarding the legal and medical requirements of tissue donation, 948 (80.1%) participants thought that tissue donation requires the same legal and medical procedures as organ donation. When asked whether a healthy, living person can donate both organs and tissues, 885 (74.8%) agreed, while 299 (25.2%) disagreed (Figure 2). 

**Figure 3:**
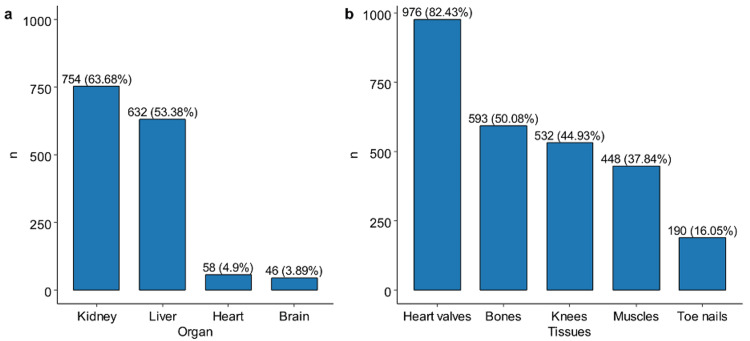
Knowledge regarding (a) organs that can be donated during life and b) tissues that can be donated after death Image generated using R software (version 4.3, R Foundation for Statistical Computing, Vienna, Austria).

The results indicated varying levels of knowledge regarding organ and tissue donation. Participants most knew that the kidney (n = 754, 63.68%) and liver (n = 632, 53.38%) are organs that could be donated during life. A small number incorrectly identified the heart (n = 58, 4.9%) and brain (n = 46, 3.89%) as organs eligible for living donation (Figure 3a). Heart valves (n = 976, 82.43%) and bones (n = 593, 50.08%) were correctly identified as tissues that could be donated after death (Figure 3b). However, knee tissues (n = 532, 44.93%) and muscles (n = 448, 37.84%) were incorrectly recognized as transplantable tissues. Additionally, some participants incorrectly identified toenails (n = 190, 16.05%) as a transplantable tissue (Figure 3). 

Ethical considerations, religious beliefs, and personal beliefs

Less than one-half of the respondents (n = 513,43.3%) reported that their religious beliefs influence their views on organ and tissue donation, while 348 (29.4%) stated that they have a personal or religious belief that prevents them from donating an organ. The concept of organ donation as a form of ongoing charity (Sadaqah Jariyah) was acknowledged by 855 (72.3%) respondents. More than one-third of the respondents (n = 443, 37.4%) thought that some individuals do not deserve a transplant, and a larger proportion (n = 944, 79.7%) thought that some people deserve a transplant more than others (Table 2). 

**Table 2:** Ethical considerations, religious beliefs, and preferences for organ donation recipients ^¶^ Yes/no questions. The n (%) who responded with “yes” is shown.

	Overall
	N = 1,184
Religious and personal beliefs	
Religious beliefs influence your views on organ and tissue donation ^¶^	513 (43.3%)
Have a personal or religious belief that prevents you from donating an organ ^¶^	348 (29.4%)
Organ donation is a form of ongoing charity (Sadaqah Jariyah) in some religious concepts ^¶^	855 (72.3%)
There are some people who don’t deserve a transplant ^¶^	443 (37.4%)
Certain people deserve a transplant more than others ^¶^	944 (79.7%)
Preferences for organ donation recipients	
Age	
< 18 years	702 (59.3%)
< 40 years	339 (28.6%)
< 60 years	143 (12.1%)
Smoking status	
Not important	579 (48.9%)
Non-smokers	536 (45.3%)
Smokers	69 (5.83%)
Position in waiting list	
Not important	504 (42.6%)
Longest waiting time on list	680 (57.4%)
Sickness level	
Not important	146 (12.3%)
Most sick	1,038 (87.7%)

Regarding preferences for organ donation recipients, sickness level was the most significant criterion, as 1,038 (87.7%) preferred to donate to the most severely ill patients, and only 146 (12.3%) considered sickness level unimportant. Approximately one-half (n = 536, 45.3%) preferred non-smokers, 69 (5.83%) explicitly preferred smokers, and 579 (48.9%) did not have a specific preference. More than one-half (n = 680, 57.4%) favored recipients who had the longest waiting time on the waiting list, and 504 (42.6%) considered it not important. Regarding age, 702 (59.3%) preferred recipients under 18 years, 339 (28.6%) favored those under 40 years, and 143 (12.1%) favored those under 60 years.

**Figure 4:**
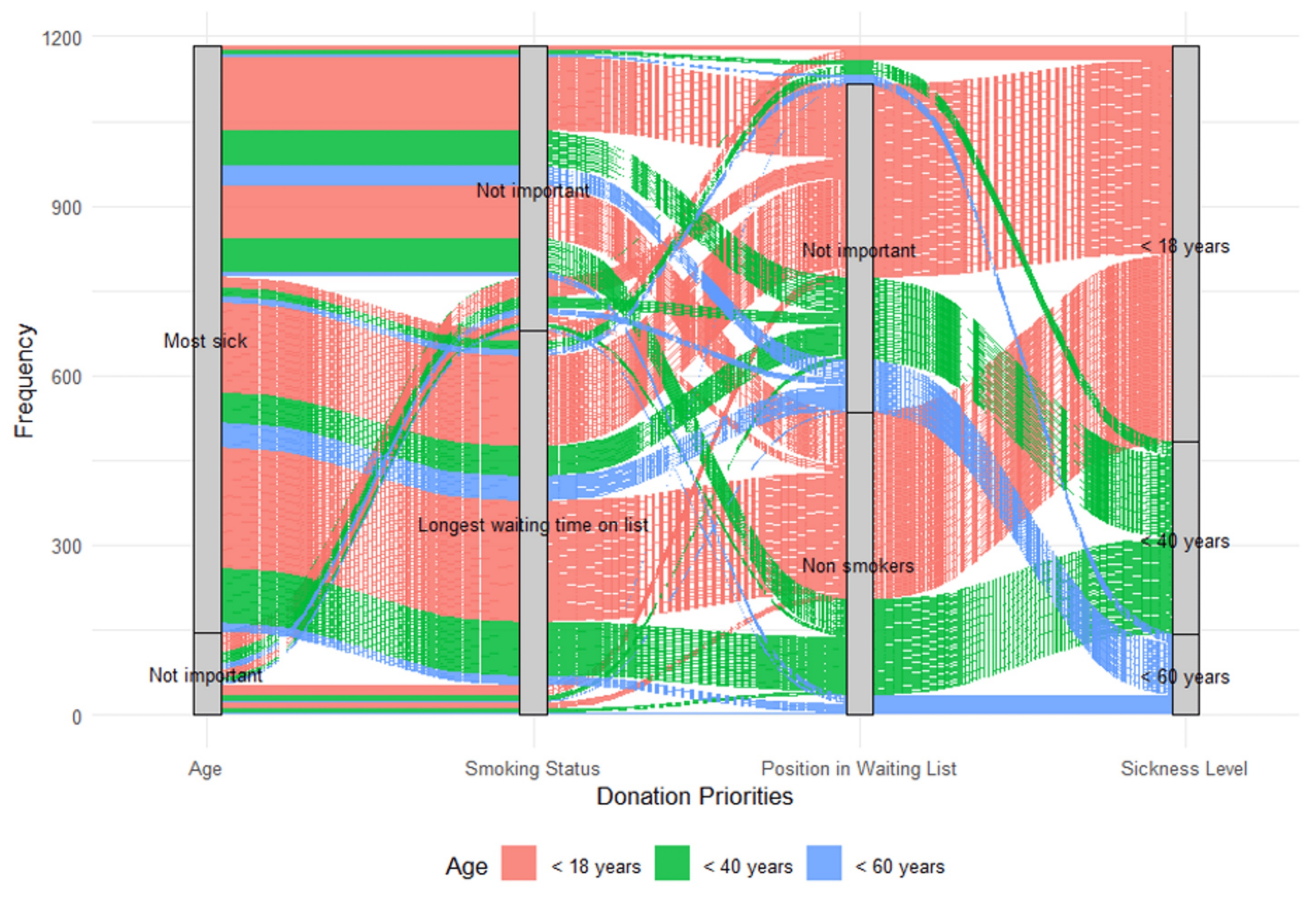
Alluvial plot of donation priorities based on recipient characteristics Image generated using R software (version 4.3, R Foundation for Statistical Computing, Vienna, Austria).

These results suggest that the strongest determining factors for prioritization were sickness level and waiting time, with younger age groups receiving higher priority (Table 3). Smoking status appeared less influential, with a considerable number of individuals indicating it was not important. Among the 1,184 individuals, the most frequently prioritized combination was the most sick, the longest waiting time on the list, age < 18 years, and non-smokers (n = 214, 18.1%). This was followed by the same category but with smoking status considered not important (n = 159, 13.4%). The third most frequent preference was most sick, not on the longest waiting list, and age < 18 years (n = 130, 11.0%). For individuals aged < 40 years, the most common preference was most sick, the longest waiting time on the list, and non-smokers (n = 96, 8.1%). This was followed by most sick, age < 18 years, and non-smokers (n = 93, 7.9%). For individuals aged < 60 years, the highest preference was most sick, the longest waiting time on the list, and smoking status considered not important (n = 44, 3.7%). The relation between the different preferences is shown in Figure 4.

**Table 3:** Combinations of recipient criteria for organ donation preferences Only the top 10 common combinations are shown.

Sickness level	Position in waiting list	Age	Smoking status	n	%
Most sick	Longest waiting time on list	< 18 years	Non-smokers	214	18.1
			Not important	186	15.7
	Not important		Not important	155	13.1
			Non-smokers	114	9.6
	Longest waiting time on list	< 40 years	Non-smokers	112	9.5
	Not important		Not important	73	6.2
	Not important		Non-smokers	65	5.5
	Longest waiting time on list		Not important	61	5.2
	Longest waiting time on list	< 60 years	Not important	54	4.6
	Not important		Not important	47	4.0

Preferences are visualized across four factors: age, smoking status, position on the waiting list, and sickness level. The width of the flows represents the frequency of participant selections, while colors indicate the preferred recipient age groups (<18 years, <40 years, and <60 years). The plot highlights the strong preference for younger, non-smoking, and severely ill recipients with the longest waiting times.

Attitudes toward organ donation and trust in the medical system​​​​​​​

One-third of the respondents (n = 435, 36.7%) did not think about the personal importance of organ and tissue donation, and 132 (11.1%) considered it not important. On the contrary, 351 (29.6%) and 266 (22.5%) were considered somewhat important and very important, respectively. Most of the participants (n = 1,086, 91.7%) believed that the public needs more information about organ and tissue donation and agreed that organs cannot be removed for donation without consent (n = 1,111, 93.8%). Nonetheless, 279 (23.6%) expressed concern that doctors and nurses might not work as hard to save the life of a critically ill patient if the individual is willing to be a donor (Table 4). 

**Table 4:** Attitudes toward organ donation and trust in the medical system ^¶^ Yes/no questions. The n (%) who responded with “yes” is shown.

	Overall
	N = 1,184
Personal importance of organ and tissue donation	
Did not think of it	435 (36.7%)
Not important	132 (11.1%)
Somewhat important	351 (29.6%)
Very important	266 (22.5%)
Public needs more information about organ and tissue donation ^¶^	1,086 (91.7%)
Organs cannot be removed from you for donation without consent ^¶^	1,111 (93.8%)
Doctors and nurses would not work as hard to save the life of a critically ill patient if he is willing to be a donor ^¶^	279 (23.6%)

Factors associated with willingness to donate​​​​​​​

Willingness to donate was significantly associated with age (p = 0.012, Cramer’s V = 0.097), with higher willingness observed among participants aged 26-35 (15.0%) and those older than 56 years (14.8%). Post-hoc pairwise comparisons with Bonferroni correction revealed that the 26-35 age group had significantly higher willingness compared to the 18-25 age group (adjusted p = 0.028), and participants aged 36-45 years were significantly more likely to consider donation very important compared to those aged 18-25 years (adjusted p = 0.008). Gender and education were not significantly associated with willingness to donate (p = 0.665 and p = 0.726, respectively). Knowing someone who was a living organ donor was significantly associated with willingness to donate (p < 0.001, Cramer’s V = 0.153), with a higher proportion of willing donors among those who knew a living donor (14.3%). Knowing someone who received an organ transplant (p = 0.009, Cramer’s V = 0.082) or a tissue transplant (p = 0.028, Cramer’s V = 0.070) also showed a statistically significant association with the willingness to donate. The personal importance of organ and tissue donation was significantly associated with willingness to donate (p < 0.001, Cramer’s V = 0.136), with willingness increasing among those who considered donation very important (13.9%). Knowing someone who needs an organ or tissue transplant was not significantly associated with willingness to donate (p = 0.238, Cramer’s V = 0.034). The source of information was not significantly associated with the willingness to donate (Table 5).

**Table 5:** Factors associated with willingness to donate Data were summarized using counts and percentages. The p-values were calculated using the chi-squared test for independence, and effect sizes were computed using Cramer’s V. Cramer’s V is a measure of association for nominal variables, ranging from 0 (no association) to 1 (perfect association). Effect sizes are interpreted as small (0.1), medium (0.3), or large (0.5).

	Not willing	Willing	P (χ²)	Cramer’s V
	N = 1,082	N = 102		
Age			0.012	0.097
18-25	642 (93.3%)	46 (6.69%)	-	-
26-35	108 (85.0%)	19 (15.0%)	-	-
36-45	147 (91.3%)	14 (8.70%)	-	-
46-55	133 (90.5%)	14 (9.52%)	-	-
>56	52 (85.2%)	9 (14.8%)	-	-
Gender			0.665	0.018
Female	703 (91.1%)	69 (8.94%)	-	-
Male	379 (92.0%)	33 (8.01%)	-	-
Education			0.726	0.045
Diploma	84 (92.3%)	7 (7.69%)	-	-
High school	420 (92.3%)	35 (7.69%)	-	-
Middle school	14 (93.3%)	1 (6.67%)	-	-
Post-graduate	52 (86.7%)	8 (13.3%)	-	-
Primary school	5 (100%)	0 (0.00%)	-	-
University	507 (90.9%)	51 (9.14%)	-	-
Know anyone who was a living organ donor			<0.001	0.153
No	747 (94.2%)	46 (5.80%)	-	-
Yes	335 (85.7%)	56 (14.3%)	-	-
Know anyone who needs an organ or tissue transplant			0.238	0.034
No	836 (92.0%)	73 (8.03%)	-	-
Yes	246 (89.5%)	29 (10.5%)	-	-
Know anyone who has received an organ transplant			0.009	0.082
No	699 (93.1%)	52 (6.92%)	-	-
Yes	383 (88.5%)	50 (11.5%)	-	-
Know anyone who has received a tissue transplant			0.028	0.070
No	973 (92.1%)	84 (7.95%)	-	-
Yes	109 (85.8%)	18 (14.2%)	-	-
Personal importance of organ and tissue donation			<0.001	0.136
Did not think of it	420 (96.6%)	15 (3.45%)	-	-
Not important	122 (92.4%)	10 (7.58%)	-	-
Somewhat important	311 (88.6%)	40 (11.4%)	-	-
Very important	229 (86.1%)	37 (13.9%)	-	-
Donation disfigures the body, preventing viewing of the body			0.149	0.042
No	736 (90.5%)	77 (9.47%)	-	-
Yes	346 (93.3%)	25 (6.74%)	-	-
Have a personal or religious belief that prevents donation			0.002	0.099
No	750 (89.7%)	86 (10.3%)	-	-
Yes	332 (95.4%)	16 (4.60%)	-	-

The perceived importance of organ and tissue donation was significantly associated with age (p = 0.011), with the highest proportion of participants considering donation very important reported by those aged 36-45 (49.5%) and >56 years (44.7%). Gender was also significantly associated with the perceived importance (p = 0.001), with a higher number of females thinking that donation is very important (36.3%) than males (33.9%). Education was not significantly associated with perceived importance (p = 0.234).

Knowing someone who was a living organ donor was significantly associated with greater perceived importance (p = 0.016), with 41.2% of them considering donation very important. Similarly, knowing someone who needs an organ or tissue transplant (p < 0.001), someone who has received an organ transplant (p < 0.001), or someone who has received a tissue transplant (p = 0.015) were associated with higher perceived importance, with the highest number of those who perceived it as very important among those with these connections (48.2%, 43.9%, and 47.5%, respectively).

Believing that organ and tissue donation disfigures the body was significantly associated with lower perceived importance (p < 0.001). Personal or religious beliefs preventing donation were also significantly associated with perceived importance (p < 0.001), with only 25.1% of those holding such beliefs considering donation very important compared to 39.3% among those without such restrictions (Table 6).

**Table 6:** Factors associated with a positive attitude toward donation Data were summarized using counts and percentages. The p-values were calculated using the chi-squared test for independence, and effect sizes were computed using Cramer’s V. Cramer’s V is a measure of association for nominal variables, ranging from 0 (no association) to 1 (perfect association). Effect sizes are interpreted as small (0.1), medium (0.3), or large (0.5).

	Not important	Somewhat important	Very important	P (χ²)	Cramer’s V
	N = 132	N = 351	N = 266		
Age				0.011	0.132
18-25	85 (19.2%)	219 (49.4%)	139 (31.4%)	-	-
26-35	17 (21.8%)	33 (42.3%)	28 (35.9%)	-	-
36-45	10 (9.71%)	42 (40.8%)	51 (49.5%)	-	-
46-55	11 (12.6%)	45 (51.7%)	31 (35.6%)	-	-
>56	9 (23.7%)	12 (31.6%)	17 (44.7%)	-	-
Gender				0.001	0.123
Female	72 (14.0%)	255 (49.7%)	186 (36.3%)	-	-
Male	60 (25.4%)	96 (40.7%)	80 (33.9%)	-	-
Education				0.234	0.082
Diploma	14 (26.9%)	18 (34.6%)	20 (38.5%)	-	-
High school	49 (16.1%)	157 (51.6%)	98 (32.2%)	-	-
Post-graduate	6 (16.2%)	17 (45.9%)	14 (37.8%)	-	-
University	63 (17.7%)	159 (44.7%)	134 (37.6%)	-	-
Know anyone who was a living organ donor				0.016	0.102
No	91 (19.9%)	221 (48.3%)	146 (31.9%)	-	-
Yes	41 (14.1%)	130 (44.7%)	120 (41.2%)	-	-
Know anyone who needs transplant				<0.001	0.153
No	107 (19.4%)	274 (49.6%)	171 (31.0%)	-	-
Yes	25 (12.7%)	77 (39.1%)	95 (48.2%)	-	-
Know anyone who has received transplant				<0.001	0.161
No	93 (20.9%)	219 (49.3%)	132 (29.7%)	-	-
Yes	39 (12.8%)	132 (43.3%)	134 (43.9%)	-	-
Know anyone who has received a tissue transplant				0.015	0.105
No	121 (18.7%)	309 (47.7%)	218 (33.6%)	-	-
Yes	11 (10.9%)	42 (41.6%)	48 (47.5%)	-	-
Donation disfigures the body, preventing viewing of the body				<0.001	0.132
No	75 (14.0%)	264 (49.4%)	195 (36.5%)	-	-
Yes	57 (26.5%)	87 (40.5%)	71 (33.0%)	-	-
Have a personal or religious belief that prevents you from donating an organ				<0.001	0.159
No	77 (14.0%)	257 (46.7%)	216 (39.3%)	-	-
Yes	55 (27.6%)	94 (47.2%)	50 (25.1%)	-	-

## Discussion

The results of the current study indicate some key issues and discrepancies in comparison to previous research studies on organ donation awareness and sources of information. A common trend in earlier studies is an inclination toward internet-based sources. In this particular study, 45.4% of participants identified the internet as their primary source, which aligns with a common method found in another study conducted among the Jordanian population [16]. This contrasts with another study, which reported television as the primary source of information, with 40% of respondents [13]. This may be explained by the growing availability of the internet and the prevalence of smartphones and social media, which serve as primary tools for information among younger populations [17,18].

The study's findings on common misconceptions about organ donation underscore the need for ongoing education programs. A significant 68.7% of participants in the current research correctly disagreed with the misconception that organ donation results in bodily disfigurement, which is consistent with the findings of an article that identified prevalent myths surrounding organ donation [19]. Nevertheless, another article found that 20.9% of participants still thought organ donation resulted in disfigurement, despite improvements, underscoring the persistent prevalence of false beliefs [20]. This persistence, particularly in areas where organ donation is not commonly discussed or encouraged in public discourse, may be explained by a lack of focused awareness initiatives, cultural beliefs, or inadequate access to accurate medical information.

More than half of the participants correctly identified that kidney and liver donations can occur while a person is still alive, demonstrating a solid understanding of transplantable organs and tissues. This reflects a strong level of knowledge about these types of donations. Interestingly, these results align with findings from Al-Kharaj City in Saudi Arabia, where an impressive 95.2% of respondents knew that kidneys could be donated, although fewer were aware that other organs, like the heart and lungs, could also be transplanted [10]. Some people were still confused, though, by tissues like knee muscles and tissues, which they mistakenly believed could be donated. This confusion likely persists due to limited public awareness of the specifics of tissue donation. This indicates the importance of providing more concentrated education on which specific organs and tissues are suitable for donation. Confusion can arise from vague information in school programs or public health campaigns, which often overlook tissue donation in favor of organ donation.

According to our results, 43.3% of respondents (n = 513) reported that their religious convictions influence their feelings about donating organs and tissues. In comparison to a study conducted in Saudi Arabia, only 19.6% of participants expressed a desire to register as an organ or tissue donor [21]. Also, the same study found that after speaking with a religious leader, 26.6% of respondents said they would think about giving their organs, and 43.9% said they would give the idea more serious thought if a reputable organization approached them [21]. In another study regarding the acceptance of organ donation, there were religious differences. For example, compared to 66% of non-Muslims, 48% of Muslims would accept an organ donation regardless of their relationship to the donor [22]. Additionally, 29.4% (n = 348) stated that they have a personal or religious belief that prevents them from donating an organ. In another study, 32.29% of respondents reported having a religious belief that prevents them from donating [20]. These results demonstrate that religious convictions have a substantial impact on attitudes toward organ donation, notwithstanding the persistence of moral prejudices and misconceptions. Many people consider donations to be a continuous act of kindness, but there is still little social acceptance of them. This is largely because religious beliefs often prioritize the sanctity of the body and emphasize moral and ethical guidelines, leading to reluctance in supporting organ donation despite its potential benefits.

Regarding preferences for organ donation recipients, the level of sickness was the most significant criterion, as 87.7% (n = 1,038) preferred to donate to patients who were most severely ill. In contrast, other research indicates that the recipient's health status is the most crucial consideration when donating an organ (68%), with saving a life being the primary rationale for organ donation (89.3%) [23]. A 2015 Australian study also favored younger patients and those with longer wait times and found that smoking negatively impacted the chances of receiving an organ [24]. Similarly, a 2022 Australian survey on kidney allocation found strong support for prioritizing the sickest and longest-waiting patients, especially children [25].

However, 15.6% of Saudi medical students in a paper mentioned that organs should be donated to a fellow religious person [19]. The findings suggest that medical urgency and fairness are the main drivers of prioritization decisions, while lifestyle factors such as smoking play a lesser role.

Our findings reflect a complex attitude toward organ and tissue donation. While some respondents had never considered its importance and a few saw it as unnecessary, more than half of our participants still consider it somewhat important or very important.

This divided perception is consistent with findings from other studies across various regions. For example, a 2023 Jazan study found that nearly half (49.2%) agreed with organ donation, and a third (33.5%) were neutral, reflecting public hesitation [20]. Almost all the participants in our study agreed that more public information is needed, which aligns with a 2016 study from the Central Region of Saudi Arabia, where 85.1% of university students believed public awareness was lacking [26]. This pattern of insufficient education is also seen worldwide, including in a 2025 Kazakhstan study highlighting poor awareness as a key barrier, reinforcing that this issue goes beyond local contexts and remains a global concern [27].

Trust in the medical system was another major concern: 23.6% of our responders worried that doctors and nurses might not try their best to save donors, a fear echoed in Argentina, where 36.4% of medical students feared being declared dead prematurely for organ retrieval, and 25.8% expressed a lack of trust in the system [28]. 

Despite strong support for promoting donation, such as the 78% approval in a 2017 Al-Kharj study, actual willingness to donate remains low (24.5%), likely linked to knowledge levels - those with better knowledge were more willing to donate, while those less knowledgeable tended to have a negative attitude [10]. Overall, while there is broad agreement on raising awareness and ensuring consent, hesitation persists due to a lack of knowledge and mistrust of the medical system. This global pattern suggests efforts should focus not only on education but also on building trust through clear policies and community involvement, which are essential to increasing both willingness and acceptance of organ donation.

The study demonstrates that willingness to donate was significantly associated with age, with higher willingness observed among participants aged 26-35 (15.0%) and those older than 56 years (14.8%). A study found that (70.5%) of participants who were willing to donate body organs were less than 30 years old [23]. 

However, gender and education were not significantly associated with willingness to donate in the current study. Surprisingly, Al-Kharaj’s study indicates a strong relationship between the male gender and the desire to donate. Additionally, they discovered another significant relationship with university educational level [10]. Another study supports the notion that the level of education in Saudi Arabia has an impact on the overall receipt of donations [29]. 

Strengths and limitations

The current research article gives a valuable perspective on how people in Makkah view organ donation, a strongly religious and culturally important city that has not been researched previously. Through the gathering of perceptions from a heterogeneous adult population, the research fills an important gap in the existing literature regarding awareness and attitudes toward organ donation among Saudi nationals.

One of the major findings of this study is the difference between people who are willing to be donors (72.7%) and those who are actually registered as donors (8.2%), highlighting a clear disconnect between intention and action. Factors associated with willingness include previous personal exposure to transplantation, religion, and age; however, there was no significant association with gender or level of education.

There are several notable strengths to this study. The sample size was far larger than the minimum required. A structured questionnaire, including key sociodemographic variables, allowed for a more comprehensive evaluation of factors associated with organ donation attitudes.

However, some limitations need to be addressed. The cross-sectional design precludes causal inference, and the use of self-reported data may contribute to social desirability bias. Additionally, an online convenience sampling method may introduce selection bias and overrepresentation of younger and more educated groups, which limits the generalizability of this study's findings. Also, data are limited to quantitative insights and do not allow for in-depth exploration of underlying beliefs and motivations. Finally, post-hoc analyses and multiple comparisons adjustment were not performed.

## Conclusions

This study provides insight into public perception regarding organ donation in Makkah, which is influenced by geographical and religious factors. While some knowledge domains were high, there are considerable gaps and misperceptions, particularly with regard to eligibility, donation processes, and trust in the healthcare system. A key finding of this study is the substantial gap between willingness to donate (72.7%) and actual donor registration (8.2%), highlighting a clear disconnect between intention and action. Factors associated with willingness included previous personal exposure to transplantation, religion, and age, with individuals who knew a living donor demonstrating significantly higher willingness to donate (14.3% vs. 5.8%; p < 0.001). However, no significant association was found with gender or education level.

The cross-sectional nature of this study means these findings should be interpreted as associations only, not causal relationships. Also, the results are to be interpreted with caution, as non-probability sampling was used and selection bias may have occurred.Together, these findings emphasize the importance of targeted educational strategies, enhanced public awareness initiatives, and collaboration with healthcare workers and religious authorities to clarify misconceptions and foster trust. Such methods may help improve public engagement and might increase organ donation rates.

## Appendices

Questionnaire Title: Exploring Public Perspectives on Organ Donation in Makkah, Saudi Arabia 

Introduction

We are a research group from the College of Medicine, Umm Al-Qura University. This questionnaire aims to explore public perspectives on organ donation in Makkah, Saudi Arabia.

Participation in this study is completely voluntary. All responses will remain strictly confidential and will be used for research purposes only. The questionnaire will take approximately five minutes to complete.

This study is conducted under the supervision of Dr. Nada Bajuaifer and has been approved by the Umm Al-Qura University Research Ethics Committee.

For inquiries, please contact nabajuaifer@uqu.edu.sa.

Thank you for your participation.

Section 1: Consent 

1. Do you agree to participate in this study? 

☐ Yes ☐ No 

Section 2: General Information 

2. Are you a resident of Makkah? 

☐ Yes ☐ No 

3. Data collector number: ______ 

*Section 3: Demographic Information* 

4. Age: 

☐ <18 

☐ 18-25 

☐ 26-35 

☐ 36-45 

☐ 46-55 

☐ 56-65 

☐ >65 

5. Gender: 

☐ Male ☐ Female 

6. Nationality: 

☐ Saudi ☐ Non-Saudi 

7. Education level: 

☐ Uneducated 

☐ Primary 

☐ Intermediate 

☐ Secondary 

☐ Diploma 

☐ Bachelor’s 

☐ Postgraduate 

Section 4: Willingness for Organ Donation 

8. Do you have an organ donor card? 

☐ Yes ☐ No 

9. Have you signed an organ donor card? 

☐ Yes ☐ No 

10. Do you have a living will? 

☐ Yes ☐ No 

11. Are your family members aware of your wishes regarding organ donation? 

☐Yes ☐ No 

Section 5: Knowledge about Organ Donation 

12. Can a healthy living person donate organs/tissues? 

☐ Yes ☐ No 

13. Can a healthy person donate part of their liver? 

☐ Yes ☐ No 

14. Can a healthy person donate their heart? 

☐ Yes ☐ No 

15. Can a healthy person donate their brain? 

☐ Yes ☐ No 

16. Can a healthy person donate a kidney? 

☐ Yes ☐ No 

Section 6: Knowledge About Tissue Donation After Death

17. Which of the following tissues can be donated after death? (Select all that apply) 

☐ Muscles 

☐ Bones 

☐ Heart valves 

☐ Toenails 

☐ Knees 

18. Does tissue donation require the same legal and medical procedures as organ donation? 

☐Yes ☐ No 

Section 7: Social Exposure and Experience 

19. Do you think organ/tissue donation disfigures the body? 

☐ Yes ☐ No 

20. Do you know someone who has undergone tissue transplantation?

☐ Yes ☐ No 

21. Do you know someone who has undergone organ transplantation?

☐ Yes ☐ No 

22. Do you know someone who donated organs while alive? 

☐ Yes ☐ No 

23. Do you know someone who needs an organ/tissue transplant?

☐ Yes ☐ No 

24. Would you consider being a living donor for a family member or close friend? 

☐ Yes ☐ No 

25. Have you discussed organ donation with your family? 

☐Yes ☐ No 

Section 8: Personal and Religious Beliefs 

26. Do your religious beliefs influence your views on organ donation? 

☐Yes ☐ No 

27. Do you have personal/religious beliefs that prevent you from donating? 

☐ Yes ☐ No 

28. Do you believe organ donation is considered a continuous charity (Sadaqah Jariyah)? 

☐ Yes ☐ No 

Section 9: Prioritization and Attitudes 

29. Do you think some people deserve organ transplants more than others? 

☐ Yes ☐ No 

30. Who do you think should be prioritized? 

☐ Younger age 

☐ Less than 40 

☐ Less than 60 

31. Smoking status priority: 

☐ Smoker 

☐ Non-smoker 

☐ Does not matter 

32. Priority based on waiting time: 

☐ Longest waiting time 

☐ Does not matter 

33. Priority based on severity: 

☐ Most severely ill 

☐ Does not matter 

Section 10: General Perceptions and Sources 

34. How important is organ/tissue donation to you?

☐ Not important

☐ Somewhat important 

☐ Very important 

☐ Never thought about it 

35. Do you think the public needs more awareness about organ donation? 

☐ Yes ☐ No 

36. After death, organs cannot be removed without consent? 

☐ True ☐ False 

37. Doctors may not try hard to save patients who are organ donors? 

☐ True ☐ False 

38. Do you think wealthy people receive transplants earlier than others? 

☐ Yes ☐ No 

39. What is your main source of information about organ donation?

☐ TV 

☐ Radio 

☐ Internet 

☐ Friends 

☐ Family 

☐ Newspapers 

☐ No specific source 
